# Comparative Analysis of the Impact of Protein on Virus Retention for Different Virus Removal Filters

**DOI:** 10.3390/membranes14070158

**Published:** 2024-07-17

**Authors:** Mohammad A. Afzal, Joshua Peles, Andrew L. Zydney

**Affiliations:** Department of Chemical Engineering, Pennsylvania State University, University Park 16802, PA, USA; maa6831@psu.edu (M.A.A.); jmp6368@psu.edu (J.P.)

**Keywords:** virus filtration, protein fouling, monoclonal antibody, virus clearance

## Abstract

The performance of virus filters is often determined by the extent of protein fouling, which can affect both filtrate flux and virus retention. However, the mechanisms governing changes in virus retention in the presence of proteins are still not well understood. The objective of this work was to examine the effect of proteins on virus retention by both asymmetric (Viresolve^®^ NFP and Viresolve^®^ Pro) and relatively homogeneous (Ultipor^®^ DV20 and Pegasus^TM^ SV4) virus filtration membranes. Experiments were performed with bacteriophage ϕX174 as a model parvovirus and human serum immunoglobulin G (hIgG) as a model protein. The virus retention in 1 g/L hIgG solutions was consistently less than that in a protein-free buffer solution by between 1 to 3 logs for the different virus filters. The virus retention profiles for the two homogeneous membranes were very similar, with the virus retention being highly correlated with the extent of flux decline. Membranes prefouled with hIgG and then challenged with phages also showed much lower virus retention, demonstrating the importance of membrane fouling; the one exception was the Viresolve^®^ Pro membrane, which showed a similar virus retention for the prefouled and pristine membranes. Experiments in which the protein was filtered after the virus challenge demonstrated that hIgG can displace previously captured viruses from within a filter. The magnitude of these effects significantly varied for the different virus filters, likely due to differences in membrane morphology, pore size distribution, and chemistry, providing important insights into the development/application of virus filtration in bioprocessing.

## 1. Introduction

Mammalian cell-based expression systems are increasingly used for the production of recombinant protein products. For example, Chinese hamster ovary cells are the most commonly employed cell line for the manufacture of monoclonal antibodies [1]. However, these mammalian cell systems are susceptible to virus contamination, including by the parvovirus minute virus of mice (MVM) [2]. The risks of virus contamination can be dramatically reduced by utilizing low-risk starting materials and testing in-process streams, but there is still a need to incorporate effective methods of virus inactivation and/or removal as part of the downstream purification process [3,4,5]. Virus filtration is widely accepted across the industry as a robust, size-based method for the removal of virus particles [6].

A number of studies have demonstrated that the virus removal capabilities of commercial virus filters can be significantly altered by the presence of a therapeutic protein. Regulatory agencies thus require that virus filter challenges be performed in the actual process intermediates [7]. For instance, Stuckey et al. [8] evaluated virus retention for the Planova^TM^ 20N virus removal filter using 11 different process streams, with the log reduction value (LRV) ranging 2.86 to 7.15. There was no apparent correlation with the pH, product concentration, solution conductivity, physical characteristics of the product, or extent of fouling (as measured by the flux decay). In contrast, corresponding challenge experiments performed with the XMuLV retrovirus in the same process streams showed undetectable levels of virus in all permeate samples (LRV ≥ 6). Bolton et al. [9] noted a significant reduction in virus retention with process throughput for the Viresolve^®^ NFP membrane, with a much greater decline in LRV for virus challenge experiments performed in the presence of proteins. The measured LRV was highly correlated with the extent of flux decline, suggesting that the primary effect of the protein was due to protein fouling, which was thought to cause a shift in flow due to the preferential blockage of the smaller pores within the filter.

Suh et al. [10] examined the retention of the MS2 bacteriophage by seven commercial virus filters using bovine serum albumin (BSA) as a model protein. The Viresolve^®^ NFP membrane showed the greatest flux decline and the greatest reduction in LRV, with the virus retention decreasing to LRV ≈ 1 after only 70 L/m^2^. The Pegasus^TM^ SV4 membrane also showed a significant reduction in LRV, even though the flux decline for this filter was minimal. Virus breakthrough was not seen when the filters were prefouled with BSA, suggesting that the effects of proteins on virus retention are not simply due to flux decline. Subsequent work [11] showed that the Viresolve^®^ Pro membrane provided the robust retention of the PP7 bacteriophage even under highly fouling conditions. Lute et al. [12] identified several instances of virus breakthrough for certain virus filters even in the absence of any significant flux decay, indicating that protein fouling is not a universally reliable predictor of viral clearance. Hongo-Hirasaki et al. [13] reported that PPV retention through the Planova^TM^ 20N was unaffected by IgG concentration. Likewise, Leisi et al. [14] observed that MVM retention by the Planova^TM^ 20N membrane was largely unaffected by protein fouling, but there was a significant reduction in virus retention for the Pegasus^TM^ SV4 membrane when operated under highly fouling conditions. Afzal and Zydney [15] also found a large reduction in virus retention by the Pegasus^TM^ SV4 membrane when challenged with solutions of human serum immunoglobulin G (hIgG) spiked with the bacteriophage ϕX174, with the data suggesting that the hIgG displaced previously captured ϕX174 from within the pores of this membrane. Kosiol et al. [16] demonstrated that hIgG could cause virus aggregation, particularly at low pH, leading to an increase in virus retention under these conditions. However, the retention of the PP7 bacteriophage at pH 9 was reduced in the presence of hIgG, which the authors attributed to competitive adsorption effects, with hIgG occupying binding sites that would otherwise capture PP7. 

Further support for the presence of interactions between viruses, proteins, and membranes is provided by studies showing a shift in the location of virus capture in the presence of proteins. For example, Adan-Kubo et al. [17] showed that fluorescently labeled B19V were captured closer to the inner surface of the Planova^TM^ 20N membrane in the presence of proteins, with the magnitude of this shift being greatest for experiments performed with albumin relative to antithrombin, haptoglobin, or hIgG. Similar results were obtained by Leisi et al. [14] for the Planova^TM^ 20N and BioEX membranes using fluorescently labeled MVM in the presence of hIgG, but the MVM were captured farther into the depth of the Pegasus^TM^ SV4 membrane. The authors hypothesized that this unusual behavior for the Pegasus^TM^ SV4 membrane was due to competition between the protein and virus for capture sites within the filter.

Additional insights into the impact of protein fouling on virus retention have been obtained by “prefouling” membranes through the filtration of a protein solution before challenging the membranes with a protein-free solution of the virus. Afzal and Zydney [15] demonstrated that prefouling the Pegasus^TM^ SV4 membrane with hIgG led to a 1-log reduction in virus capture that was nearly independent of the initial degree of fouling. Khan et al. [18] observed no change in virus retention when prefouling the Viresolve^®^ NFP membrane with BSA to 30% flux decay, but they found an increase in virus retention for membranes fouled to 60% flux decay. Jackson et al. [19] found no impact on virus retention after prefouling the Ultipor^®^ DV20 membrane with hIgG up to a 50% flux decay, which was similar to results obtained by Suh et al. [10] for the Viresolve^®^ Pro and Pegasus^TM^ SV4 membranes prefouled with BSA. The origin of these differences in performance is unclear.

The objective of this study was to obtain more detailed insights into the impact of proteins on virus retention through four different commercial virus filtration membranes: the asymmetric Viresolve^®^ NFP and Viresolve^®^ Pro membranes and the relatively homogeneous Ultipor^®^ DV20 and Pegasus^TM^ SV4 membranes. Data were obtained for filters challenged with a model virus both in the presence and absence of hIgG and after prefouling the membranes with virus-free protein solutions. Experiments were also performed in staged filtration experiments to identify the effect of proteins on previously captured viruses. These results provide new insights into how proteins can alter the virus retention characteristics of different virus removal filters.

## 2. Materials and Methods

### 2.1. Virus Filters

Virus challenges were performed with a single layer of four commercially available virus filtration membranes: the highly asymmetric Viresolve^®^ NFP and Viresolve^®^ Pro membranes from MilliporeSigma Corporation (Bedford, MA, USA) and the relatively homogenous Ultipor^®^ DV20 and Pegasus^TM^ SV4 membranes from Cytiva (Marlborough, MA, USA), as summarized in Table 1. Single layers of membrane were used in all experiments to make it easier to accurately measure the virus retention without having to use very highly concentrated suspensions of bacteriophages.

The Viresolve^®^ NFP and Viresolve^®^ Pro membranes were obtained as single-layer sheets directly from the manufacturer. The other two membranes were provided as 47 mm dual-layer discs, which were carefully separated into individual layers by immersing them in deionized water. Then, 25-mm discs were cut and placed in a polypropylene filter holder (Advantec MFS Inc., Dublin, CA, USA), providing a filtration area of 3.5 cm^2^, with the skin/shiny side facing away from the feed (the orientation used in the commercial virus filtration devices). Limited experiments were conducted using the Viresolve^®^ NFP membrane in an Amicon^®^ Model 8010 ultrafiltration cell and using the Viresolve^®^ Pro membrane in a custom Micro 40 device provided by MilliporeSigma (Bedford, MA, USA), both with a single layer of the membrane. 

Filters were fouled with solutions of human serum immunoglobulin G (hIgG), obtained from Nova Biologics (Oceanside, CA, USA) as a lyophilized powder that was then mixed with 0.10 M phosphate buffered saline (PBS) to obtain the desired hIgG concentration. The hIgG was filtered first through 0.2 µm and then through 0.1 µm pore size filters (Cytiva, Marlborough, MA, USA) to remove any large protein aggregates prior to use.

### 2.2. Bacteriophage

Bacteriophage ϕX174 (ATCC-13706-B1, Manassas, VA, USA), which has been extensively used as a surrogate for mammalian viruses, was propagated in host *E. coli* (ATCC13706, Manassas, VA, USA) by mixing 100 μL of phage with *E. coli* grown in nutrient broth (NB) media prepared by mixing 8 g/L of nutrient broth powder (BD-234000) and 5 g/L of NaCl (Promega Corp., Madison, WI, USA) with deionized distilled water. The phages were propagated at 37 °C for ~6 h with gentle agitation in an incubator shaker (New Brunswick Scientific, Edison, NJ, USA). The phage suspension was centrifuged at 3500 rpm for 10 min (Beckman Coulter, Brea, CA, USA), with the phage collected in the supernatant and then filtered through a 0.2 µm sterile filter. 

ϕX174 concentrations were determined using a plaque-forming assay by mixing 100 μL of the phage samples with 200 μL of *E. coli* and 3 mL of soft agar, which were then poured onto hard agar plates that were incubated at 37 °C for approximately 6 h in an inverted position. The number of plaques was determined by visual counting, with samples diluted to obtain a countable number of plaques on each plate (typically from 5 to 200). Additional details on the handling and analysis of the ϕX174 are provided by Afzal and Zydney [20].

### 2.3. Virus Filtration

Virus filtration was performed at both a constant filtrate flux, which was obtained by connecting a peristaltic pump (Masterflex, Vernon Hills, IL, USA) between the feed solution reservoir and the filter holder, and a constant transmembrane pressure, which was provided by connecting the filter holder to an air-pressurized feed reservoir (SR-TEK Ltd., Milton Keynes, UK). Limited experiments were conducted with the virus filter directly fed by a syringe pump (KD Scientific, Holliston, MA, USA) to maintain a constant flux. 

The membranes were initially flushed with around 70 L/m^2^ of 100 mM PBS at pH 7.4 to thoroughly wet the pore structure. The membrane’s permeability was then evaluated from the slope of experimental data for the filtrate flux as a function of transmembrane pressure (TMP), using data at a minimum of three TMP, with the range of values for the different membrane samples provided in Table 1. 

The feed reservoir was then filled with the challenge solution: a suspension of bacteriophage ϕX174, an hIgG solution, or an hIgG solution spiked with ϕX174. The virus retention data were typically reported in terms of the log reduction value (*LRV*):

(1) $$LRV=-log_{10}\left(\frac{C_{filtrate}}{C_{Feed}}\right)$$

where *C_filtrate_* and *C_Feed_* are the ϕX174 concentrations in grab samples of the filtrate and feed solutions, respectively.

## 3. Results and Discussion

**Figure 1:**
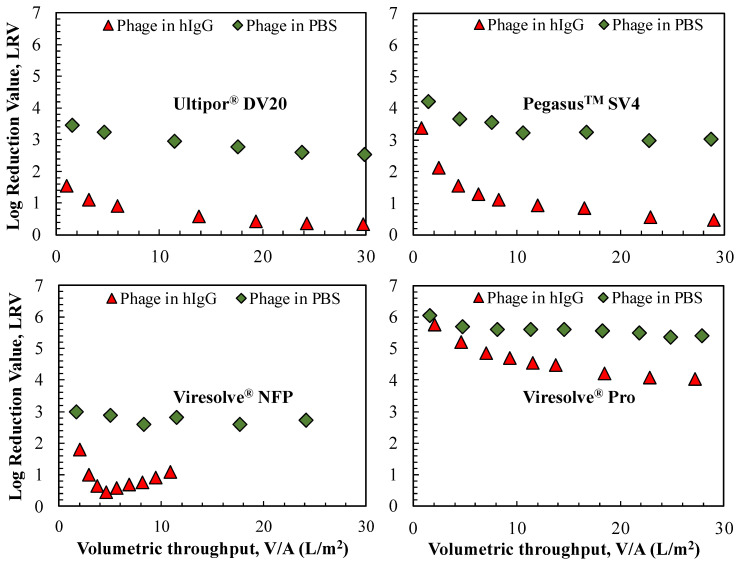
Virus retention during constant pressure filtration at 210 kPa for single layers of the Ultipor^®^ DV20, Pegasus^TM^ SV4, Viresolve^®^ NFP, and Viresolve^®^ Pro membranes for ϕX174 challenges using PBS or 1 g/L hIgG solutions. Data from single experiments; replicates are shown in Figure S1.

Figure 1 shows the data obtained during constant pressure filtration at 210 kPa (30 psi) through the four virus removal filters for the ϕX174 challenges in PBS and 1 g/L hIgG solutions, all at pH 7.4 with 150 mM ionic strength. The ϕX174 feed concentration was 10^6^ pfu/mL for the Viresolve^®^ NFP, Ultipor^®^ DV20, and Pegasus^TM^ SV4 membranes, and it was 10^8^ pfu/mL for the Viresolve^®^ Pro membrane; the higher phage concentration used for the Viresolve^®^ Pro membrane was required to obtain detectable phage concentrations in the permeate samples obtained with this highly retentive virus filter. Limited data obtained over a range of phage concentrations indicated that the measured virus retention was independent of the phage concentration, in good agreement with previous results for the Ultipor^®^ DV20 membrane [19]. Repeat experiments showed good reproducibility, with LRV values within ±0.5 and flux decline within ±10% (see Figure S1).

The filtrate flux remained nearly constant for the experiments using ϕX174 in PBS with a less than 10% decline in flux (except for the Viresolve^®^ NFP membrane, which showed closer to a 20% flux decline). In contrast, there were significant flux declines for the experiments with ϕX174 in the presence of hIgG, with a more than a 90% flux decline for the two asymmetric membranes and a more than 50% flux decline for the two homogeneous membranes. This behavior is discussed in more detail subsequently. 

The measured virus retention in grab samples obtained over the course of the constant pressure filtration is shown in Figure 1 as a function of the volumetric throughput, equal to the cumulative filtrate volume divided by the filter area. The virus retention for the commercial virus filters would all be significantly higher than the LRV shown in Figure 1 since the Ultipor^®^ DV20, Pegasus^TM^ SV4, and Viresolve^®^ Pro membranes are used as two-layers membranes in series and the Viresolve^®^ NFP membrane is sold in a three-layer configuration. The decrease in LRV for the ϕX174 in PBS has previously been attributed to the accumulation of mobile viruses within the filter, a phenomenon referred to as internal polarization [15,19]. In all cases, the LRV obtained in the presence of hIgG was lower than that obtained in PBS, similar to results reported previously for the Viresolve^®^ NFP [9] and Pegasus^TM^ SV4 membranes [15]. This effect was relatively small for the Viresolve^®^ Pro membrane, with the LRV in the presence of hIgG being about 1.3 logs smaller than that in PBS, but it was much more pronounced for the relatively homogeneous membranes; the LRV in the presence of hIgG approached zero for both the Ultipor^®^ DV20 and Pegasus^TM^ SV4 membranes, which is nearly 3 logs smaller than the values obtained in PBS. The ϕX174 retention for the Viresolve^®^ NFP membrane showed an unusual behavior, with the LRV rapidly dropping to a value of 0.45 after the filtration of only 5 L/m^2^ before increasing by about 0.5 logs over the subsequent 5 L/m^2^. This may reflect the development of a filter cake or extensive pore blockage at very high degrees of fouling, which would cause an increase in ϕX174 retention during the latter stages of filtration. Khan et al. [18] presented a similar hypothesis for the increase in virus retention observed with viral stock solutions containing high levels of impurities.

**Figure 2:**
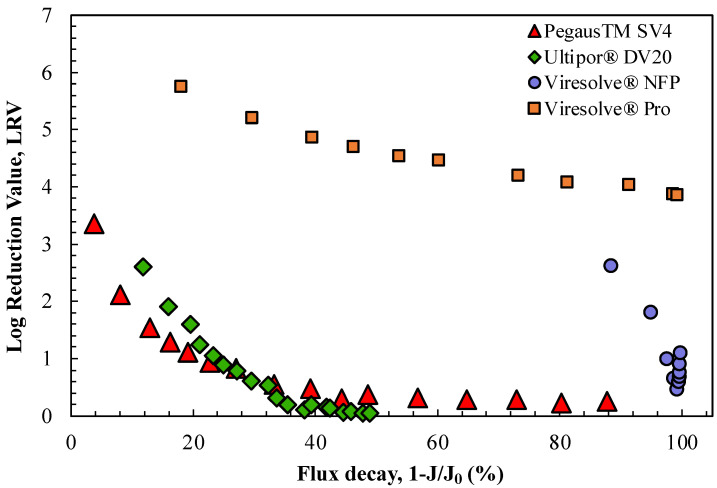
LRV as a function of flux decline for the experimental data from Figure 1.

Bolton et al. [9] previously hypothesized that the decline in LRV observed for the Viresolve^®^ NFP membrane in the presence of proteins was directly related to the magnitude of the flux decline caused by protein fouling. This was examined for the four virus filters by replotting the data in Figure 1 as an explicit function of 1-J/J_o_, where the values of the initial filtrate flux for each membrane (J_o_) were evaluated based on data for the buffer flux at the same TMP. The results are shown in Figure 2; data from replicate experiments are plotted in Figure S2. The data for the two relatively homogeneous membranes, the Ultipor^®^ DV20 and Pegasus^TM^ SV4 membranes, tend to collapse to nearly a single curve when plotted in this fashion, although the virus retention decreased to nearly zero after only a 40% flux decline. This decline in LRV was likely related to the accumulation of ϕX174 in the capture zone of these homogeneous membranes in combination with the elimination of capture sites within the filter by the hIgG, either due to protein adsorption and/or the blockage of the size-restrictive pores. In contrast, the two highly asymmetric membranes, the Viresolve^®^ Pro and Viresolve^®^ NFP membranes, both showed very high degrees of fouling, but the virus retention for the Viresolve^®^ Pro membrane remained at LRV > 4 while that for the Viresolve^®^ NFP membrane sharply decreased, with the latter in good agreement with results obtained by Bolton et al. [9]. Note that the first data point for the Viresolve^®^ NFP membrane is plotted at a flux decay of 88%, reflecting the very high degree of fouling that was obtained within the first grab sample (0.6 mL). The very high degree of fouling for the Viresolve^®^ Pro and Viresolve^®^ NFP membranes was a direct result of the highly asymmetric pore structure in these membranes, with all the protein fouling localized to the region with the highly restricted pores near the filter exit [21]. Note that the minimum in the LRV for the Viresolve^®^ NFP membrane near the very end of the experiment occurred at a flux decline of 99.3%, which is a much higher degree of fouling than is typically studied in virus filter testing.

Although most previous studies have attributed reductions in virus retention in the presence of proteins simply to the effects of protein fouling (and the resulting change in filtrate flux and pore size distribution), Afzal and Zydney [15] recently showed that hIgG could directly affect virus capture/retention by the Pegasus^TM^ SV4 membrane. This was experimentally examined by performing virus challenge experiments at a constant filtrate flux (to eliminate the effect of flux decline on virus retention) for (a) clean membranes using ϕX174 in PBS, (b) membranes prefouled with hIgG and then challenged with ϕX174 in PBS, and (c) membranes challenged with a mixture of ϕX174 and hIgG. Given the very different permeabilities and fouling characteristics for the virus filters, these experiments were performed under slightly different conditions: the Ultipor^®^ DV20 and Pegasus^TM^ SV4 membranes were both operated at a flux of 20 LMH using 3 and 5 g/L hIgG solutions, respectively, the Viresolve^®^ Pro membrane was run at 100 LMH with 0.25 g/L of hIgG, and the Viresolve^®^ NFP membrane was run at 250 LMH with 0.05 g/L of hIgG. The prefouling was performed until the transmembrane pressure increased by a factor of 4 (i.e., until P/P_o_ = 4) for the Viresolve^®^ Pro membrane and by a factor of 2.5 for the Pegasus^TM^ SV4, Ultipor^®^ DV20, and Viresolve^®^ NFP membranes. The lower degree of fouling used for the experiments with the Pegasus^TM^ SV4 and Ultipor^®^ DV20 membranes was required because of the low permeability (and thus high initial transmembrane pressure) of these membranes. The experiments with the Viresolve^®^ Pro membrane were conducted with a syringe pump to ensure that the filtrate flux remained constant.

**Figure 3:**
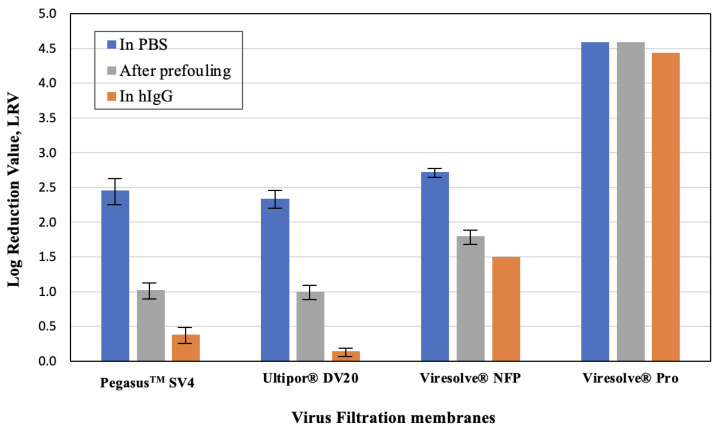
Virus retention for the different virus filtration membranes for single constant flux filtration experiments performed with ϕX174 in PBS through both clean and prefouled membranes and with ϕX174 in hIgG solutions. Prefouling was performed with the constant flux filtration of hIgG through the filters until P/P_0_ = 2.5 for the Viresolve^®^ NFP, Pegasus^TM^ SV4, and Ultipor^®^ DV20 membranes and to P/P_0_ = 4 for the Viresolve^®^ Pro membrane. Error bars represent the range of values for replicate experiments.

The measured values of ϕX174 in the grab samples obtained at a throughput of 20 L/m^2^ are summarized in Figure 3. In all cases, the highest virus retention was obtained for the clean membranes, while the lowest virus retention was obtained for the experiments in which the ϕX174 challenge was performed in the presence of hIgG, even though the experiments performed using the hIgG began with membranes that were “unfouled” and the pressure at a throughput of 20 L/m^2^ was always less than or equal to that for the corresponding prefouled membrane. The difference in LRV between that in the prefouled membranes and that evaluated in the presence of hIgG was particularly pronounced for the relatively homogeneous Pegasus^TM^ SV4 and Ultipor^®^ DV20 membranes, with a more than 0.7-log difference in LRV. These homogeneous membranes show virus capture in a relatively larger portion of the filter depth [14,19,22], with the data suggesting that hIgG may out-compete ϕX174 for these capture sites. In contrast, the LRVs for the highly asymmetric Viresolve^®^ NFP and Viresolve^®^ Pro membranes were similar for the prefouled membranes and that evaluated in the presence of hIgG, with the Viresolve^®^ Pro membrane showing robust virus retention for all three conditions. The origins of these differences in behavior are discussed in more detail subsequently.

**Figure 4:**
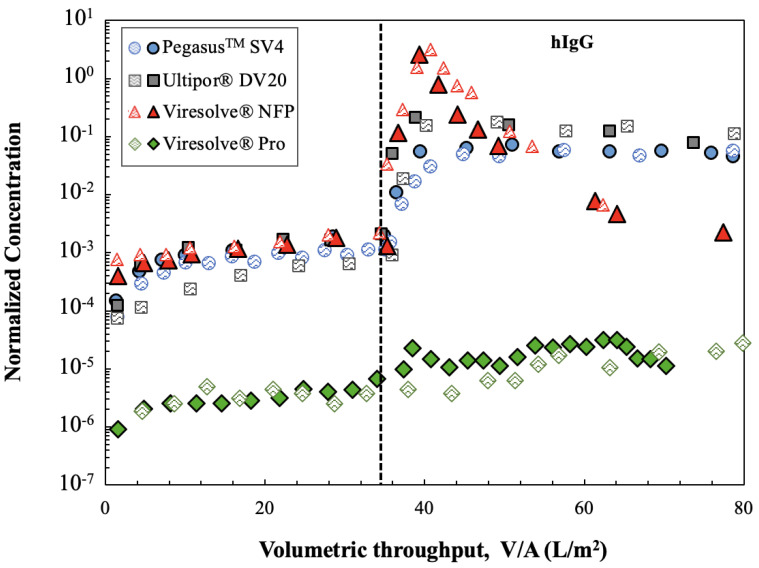
Normalized ϕX174 concentration (permeate concentration/initial feed concentration) as a function of volumetric throughput for replicate experiments involving different membranes for the filtration of ϕX174 followed by a 1 g/L hIgG solution at a constant pressure of 210 kPa. The feed was switched between ϕX174 and virus-free hIgG at 35 L/m^2^ through a trivalve, as indicated by the dashed vertical line. Results from replicate experiments are shown by the shaded and filled symbols.

Additional insights were obtained by challenging the membranes with ϕX174 in PBS followed immediately by a virus-free 1 g/L hIgG solution, all at a constant pressure of 210 kPa. A trivalve was used to switch between the ϕX174 and hIgG to ensure that there was no disruption in the pressure/flow while switching between the feed solutions. The results from replicate experiments performed with each of the virus filters are plotted in Figure 4 as the normalized ϕX174 concentration, i.e., the ratio of the ϕX174 concentration in the permeate grab sample to that in the initial feed used for the phage challenge. The phage retention levels for the Ultipor^®^ DV20, Pegasus^TM^ SV4, and Viresolve^®^ NFP membranes were nearly identical during the initial phage challenge, with the normalized ϕX174 concentration increasing by almost 1 log over the 35 L/m^2^. In contrast, the Viresolve^®^ Pro membrane had approximately 100-fold (2 logs) greater retention, although the normalized ϕX174 concentration also increased by nearly 1 log for this filter during the initial phage challenge.

**Figure 5:**
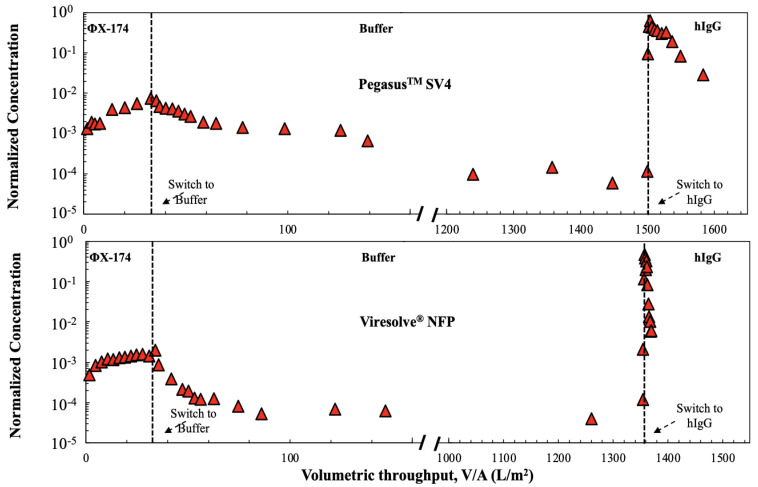
Normalized ϕX174 concentration in permeate samples collected through the Pegasus^TM^ SV4 (**top panel**) and Viresolve^®^ NFP (**bottom panel**) membranes during a 3-stage filtration process in which the filter was challenged first with ϕX174 in PBS followed by pure PBS (no phage) and then a virus-free hIgG solution, all at a constant transmembrane pressure of 210 kPa. The feed was switched between the different solutions through a trivalve, as indicated by the vertical dashed lines. Note the broken *x*-axis.

The introduction of hIgG caused a dramatic increase in the ϕX174 concentration in the permeate samples obtained through the Ultipor^®^ DV20, Pegasus^TM^ SV4, and Viresolve^®^ NFP membranes, even though the hIgG solution contained no virus. Data obtained using the same experimental system but with PBS in the second half of the filtration showed no increase in ϕX174 concentration after switching from the phage suspension to pure buffer (shown subsequently in Figure 5). The increase in virus transmission upon switching to a hIgG solution was discussed by Afzal and Zydney [15] for the Pegasus^TM^ SV4 membrane and was attributed to the displacement of previously captured phages by the hIgG. The ϕX174 concentration in the permeate samples for the Ultipor^®^ DV20 and Pegasus^TM^ SV4 membranes remained nearly constant during the hIgG challenge, with values that were approximately 100 times greater than that evaluated immediately before the introduction of the hIgG solution. The behavior of the Viresolve^®^ NFP membrane was somewhat different, with an initial 1000-fold increase in the ϕX174 concentration followed by a very significant decline in permeate concentration over the course of the hIgG filtration. This reduction in phage concentration is likely related to the very rapid fouling of the Viresolve^®^ NFP membrane by the hIgG solution, with the flux decreasing by more than 90% after only 3 L/m^2^. In contrast, the ϕX174 retention by the Viresolve^®^ Pro membrane was nearly unaffected by the switch to hIgG, with the ϕX174 concentration in the permeate remaining relatively stable throughout the hIgG filtration. It is important to note that the Ultipor^®^ DV20, Pegasus^TM^ SV4, and Viresolve^®^ NFP membranes are all made of polyvinylidene fluoride (PVDF), while the Viresolve^®^ Pro membrane is made of polyethersulfone, suggesting that the differences seen in Figure 3 and Figure 4 may be related, at least in part, to the chemistry of the filters and the nature of the virus/protein/membrane interactions. 

The different behavior of the highly asymmetric Viresolve^®^ NFP and the relatively homogeneous Pegasus^TM^ SV4 membranes were further examined using a three-stage filtration process in which the filters were first challenged with a suspension containing 10^6^ pfu/mL ϕX174 in PBS for 35 L/m^2^ throughput, followed immediately by an extended flush with just PBS and then a phage-free hIgG solution (1 g/L of hIgG for the Viresolve^®^ NFP membrane and 5 g/L of hIgG for the Pegasus^TM^ SV4 membrane). The switches in feed were again performed using a trivalve to eliminate any flow disruptions, with the pressure maintained at 210 kPa throughout the experiment. The results are shown in Figure 5. The extended buffer flush caused a gradual decline in the ϕX174 concentration for the Pegasus^TM^ SV4 membrane, with the normalized phage concentration remaining around 10^−4^ over more than 1400 L/m^2^ of filtration due to the very slow washout of previously retained phage during the buffer flush [15]. The buffer flush caused a more rapid initial decline in the ϕX174 concentration for the Viresolve^®^ NFP membrane, with the normalized concentration remaining at about 4 × 10^−5^ over the remainder of the buffer flush. Although the extended buffer flush should have led to the removal of the large majority of the mobile ϕX174 that was retained within the membrane during the initial phage challenge [19], the switch to the hIgG solution still caused a rapid, nearly 4-log increase in the normalized ϕX174 concentration for both membranes despite the very different pore morphologies. This large spike in the ϕX174 transmission was likely due to the displacement of previously captured phages (that were not removed by the extended buffer flush) by the hIgG in the final stage of the filtration experiment, possibly reflecting the presence of competitive adsorptive interactions between the ϕX174/hIgG and the PVDF membranes, with adsorption of the hIgG leading to the desorption of the ϕX174.

The potential impacts of adsorptive interactions on ϕX174 retention were further examined by challenging the Viresolve^®^ NFP and Pegasus^TM^ SV4 membranes with ϕX174 at different solution pH values. Data were obtained for the Pegasus SV4 membrane in 10 mM PBS solutions to increase the effect of electrostatic interactions, with the pH adjusted by adding HCl or NaOH as needed. The results are summarized in Table 2. The initial LRV after 10 L/m^2^ of throughput for the Viresolve^®^ NFP membrane significantly increased at low pH levels, i.e., under conditions where the ϕX174 and membranes had opposite electrical charges. There was also a small increase in LRV with decreasing pH for the Pegasus^TM^ SV4 membrane, although this effect was smaller than that seen with the Viresolve^®^ NFP membrane.

**Figure 6:**
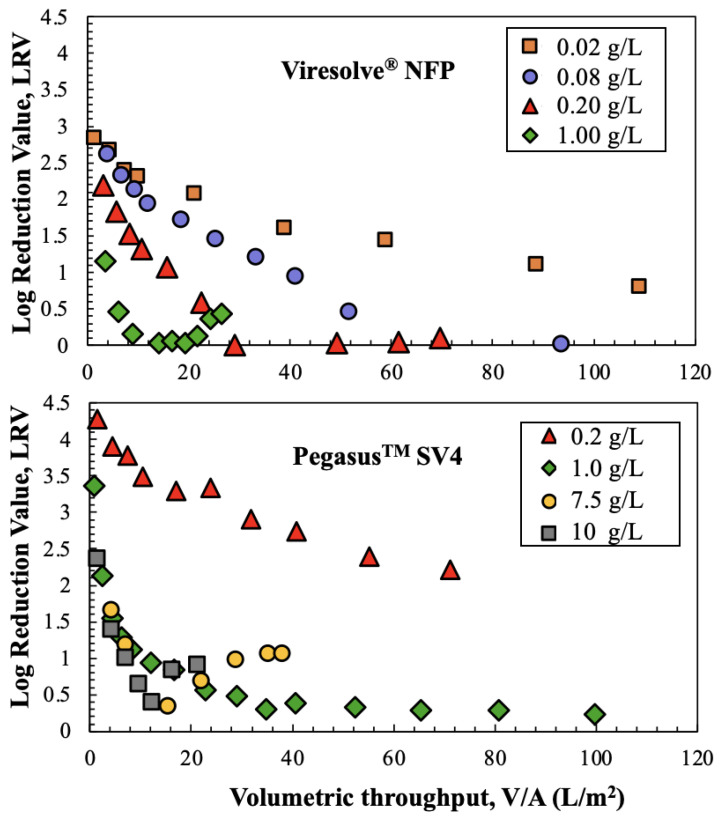
Virus retention as a function of hIgG concentration for protein solutions with 10^6^ pfu/mL of phages. Data were obtained for single experiments at each hIgG concentration involving the Viresolve^®^ NFP (**top panel**) and Pegasus^TM^ SV4 (**bottom panel**) membranes, all at a constant pressure of 210 kPa.

The effects of hIgG concentration on ϕX174 retention for the Viresolve^®^ NFP and Pegasus^TM^ SV4 membranes are examined in Figure 6. The experiments with the Viresolve^®^ NFP membrane were performed at lower hIgG concentrations due to the very high degree of fouling observed with this filter. The ϕX174 retention by the Viresolve^®^ NFP membrane decreased with increasing hIgG concentration, similar to the results presented by Bolton et al. [9], with the LRV decreasing to essentially zero for the experiments with 0.08 and 0.2 g/L hIgG solutions. Only the data at the highest hIgG concentration showed a minimum in LRV at an intermediate throughput. The data for the Pegasus^TM^ SV4 membrane showed only a 2-log reduction in LRV when the hIgG concentration was 0.2 g/L, but the LRV decline was much more pronounced for the runs at hIgG concentrations of 1 g/L or higher. In addition, the data at 7.5 and 10 g/L hIgG showed minima in the phage retention, similar to the results for the Viresolve^®^ NFP membrane at 1 g/L.

## 4. Conclusions

The results presented in this study provide detailed insights into the virus retention performance of four different commercially available virus filtration membranes. Similar to previously reported studies, virus retention was reduced in the presence of proteins compared with that obtained in a protein-free buffer. The difference in LRV was nearly 3 logs for the relatively homogenous Ultipor^®^ DV20 and Pegasus^TM^ SV4 membranes, while the highly asymmetric Viresolve^®^ Pro membrane had only about 1.3 logs lower retention (even for the heavily fouled membrane). The Viresolve^®^ NFP membrane also had a lower LRV in the presence of hIgG, but in this case, the virus retention in the presence of hIgG showed a distinct minimum, with the increase in virus retention at a very high flux decline likely due to the blockage of the pore space within the depth of the Viresolve^®^ NFP membrane (upstream of the size-selective skin layer) that provides additional resistance to transport. The LRV profiles for the Ultipor^®^ DV20 and Pegasus^TM^ SV4 membranes were well-correlated with the extent of flux decline, suggesting that this behavior is governed in large part by the relatively homogeneous structure of these two filters. 

Experiments performed with prefouled membranes at constant flux showed that only part of the reduction in LRV was due to a change in the membrane pore size distribution due to protein fouling; data for the Viresolve^®^ Pro membrane showed nearly identical virus retention levels for the pristine and prefouled membranes. The different behavior for the different virus filters is likely related to differences in the underlying pore structure/properties of the membranes, similar to the different effects of protein fouling on the location of virus capture as examined by confocal microscopy [14] and the visualization of gold nanoparticles by SEM [21]. Note that the Viresolve^®^ Pro membrane is made of polyethersulfone, while the other three virus filters examined in this work were all made of PVDF, suggesting that the more robust virus retention for the Viresolve^®^ Pro membrane may be directly related to its surface chemistry. 

Sequential filtration experiments in which the filters were first challenged with a virus and then with hIgG (without virus) showed a sharp increase in virus concentration in permeate samples obtained immediately after switching to the protein. This increase in virus transmission was due to the displacement of viruses that were previously captured within the membrane by the protein, a phenomenon that was first identified by Afzal and Zydney [15] for the Pegasus^TM^ SV4 membrane. This effect was most pronounced for the Viresolve^®^ NFP membrane and least significant for the Viresolve^®^ Pro membrane, suggesting that this behavior is not related to the asymmetric (versus homogeneous) structure of the membrane. This increase in virus transmission was also apparent even after extensively flushing the virus filter with buffer (using more than 1000 L/m^2^ of buffer). Thus, it is critically important that any pre-flushing of virus filters be performed with completely virus-free buffer solutions to ensure that the product is not contaminated with viruses that were captured during the filter preparation.

The large reduction in LRV for the Ultipor^®^ DV20, Pegasus^TM^ SV4, and Viresolve^®^ NFP membranes in the presence of proteins raises concerns about the effectiveness of these filters in providing the high level of virus removal required in bioprocessing. Similar effects have been previously reported. For example, Lute et al. [12] reported an LRV ≤ 0.5 for the Ultipor^®^ DV20 membrane using both ϕX174 and PP7 in the presence of a 2.5 mg/mL solution of a therapeutic protein, while Bolton et al. [9] found an LRV ≤ 1 for the Viresolve^®^ NFP using ϕX174 when measured in a 1 mg/mL solution of hIgG. However, it is important to note that the data obtained in this study were for single layers of membrane, while the commercially available flat-sheet virus filters typically use two or three layers of virus-retentive membranes. Peles et al. [23] showed that the hIgG fouling of the Viresolve^®^ Pro membrane occurs almost entirely in the upper layer of the two-layer filter, suggesting that virus retention by the lower layer(s) of membrane might be largely unaffected by protein fouling (but might still be affected by the displacement of viruses in the presence of proteins). In addition, the hIgG fouling of virus filters appears to be dominated by the presence of irreversible aggregates [23,24], while many monoclonal antibodies show flux decline due to the formation of reversible associations. Future studies will be required to understand the magnitude of the effect of proteins on virus retention by these multilayer filters using actual therapeutic proteins with different fouling characteristics to more fully understand how the properties of the proteins and the key morphological features of the virus filters, such as pore tortuosity, void volume fraction, pore size distribution, and pore interconnectivity, govern virus filter performance.

## Figures and Tables

**Table 1:** Summary of the virus removal filters used in this work.

Filter	Manufacturer	Polymer	Morphology	Permeability
Viresolve^®^ Pro	MilliporeSigma	Polyether- sulfone	Asymmetric	27–29 LMH/psi
Viresolve^®^ NFP	MilliporeSigma	Polyvinylidene fluoride	Asymmetric	26–32 LMH/psi
Ultipor^®^ DV20	Cytiva (formerly Pall Life Sciences)	Polyvinylidene fluoride	Relatively homogeneous	2.0–2.7 LMH/psi
Pegasus^TM^ SV4	Cytiva (formerly Pall Life Sciences)	Polyvinylidene fluoride	Relatively homogeneous	2.8–3.7 LMH/psi

**Table 2:** Effect of solution pH on virus retention by the Viresolve^®^ NFP and Pegasus^TM^ SV4 membranes.

pH	Viresolve^®^ NFP LRV	Pegasus^TM^ SV4 LRV
4.0–4.9	4.4 ± 0.2	3.1 ± 0.2
7.4	2.7 ± 0.2	2.8 ± 0.2
10	2.4 ± 0.2	2.4 ± 0.2

## Data Availability

The data presented in this study are available on request from the corresponding author.

## Supplementary Materials

The following supporting information can be downloaded at: https://www.mdpi.com/article/10.3390/membranes14070158/s1. Figure S1: Virus retention during constant pressure filtration at 210 kPa for single layers of the Ultipor^®^ DV20, Pegasus^TM^ SV4, Viresolve^®^ NFP, and Viresolve^®^ Pro membranes for replicate experiments involving ϕX174 challenges using PBS or 1 g/L hIgG solutions. Results from replicate experiments are represented as filled and shaded symbols. Figure S2: LRV as a function of flux decline for the replicate experiments involving phage in hIgG from Figure S1.
