# Supplementary material for: Comparative Analysis of the Impact of Protein on Virus Retention for Different Virus Removal Filters
Source: Membranes (Basel). 2024 Jul 17;14(7):158. doi: 10.3390/membranes14070158 (PMC11278833; doi:10.3390/membranes14070158)
Supplement: Supplementary file 1 [file membranes-14-00158-s001.zip › membranes-3084627-supplementary.pdf]

membranes

*Supplementary Materials*

# Comparative Analysis of the Impact of Protein on Virus Retention for Different Virus Removal Filters

**Mohammad A. Afzal, Joshua Peles and Andrew L. Zydney ***

Department of Chemical Engineering, Pennsylvania State University, University Park 16802, PA, USA; maa6831@psu.edu (M.A.A.); jmp6368@psu.edu (J.P.)

* Correspondence: zydney@engr.psu.edu

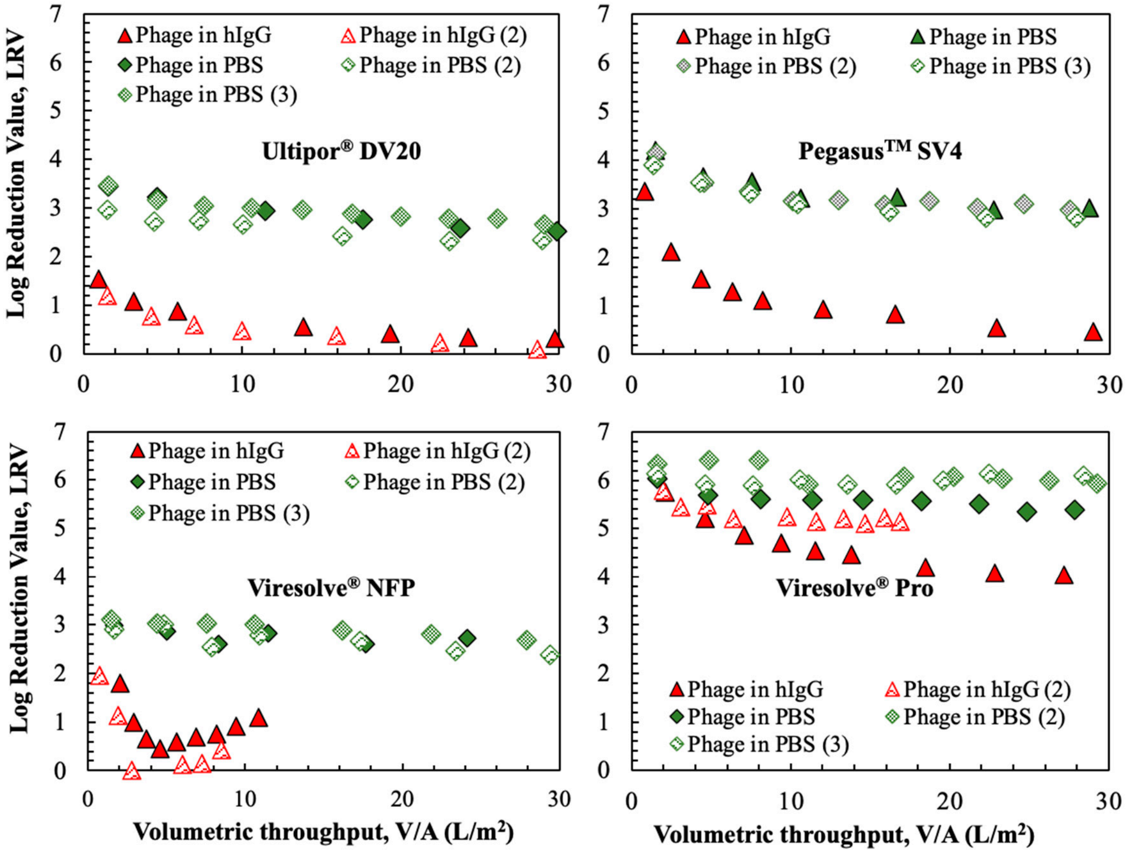


**Figure S1.** Virus retention during constant pressure filtration at 210 kPa for single layers of the Ultipor® DV20, Pegasus™ SV4, Viresolve® NFP, and Viresolve® Pro membranes for replicate experiments involving ϕX174 challenges using PBS or 1 g/L hIgG solutions. Results from replicate experiments are shown as filled and shaded symbols.

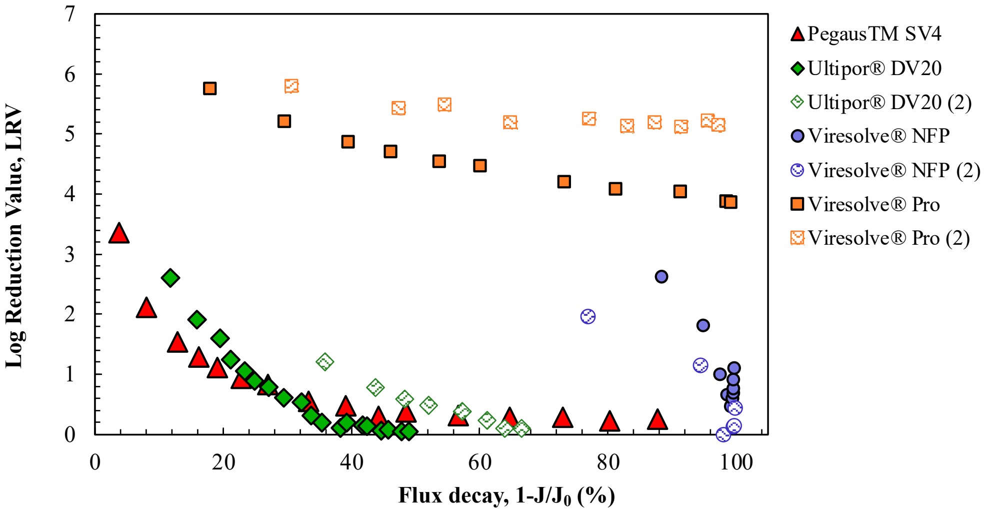


**Figure S2.** LRV as a function of flux decline for the replicate experiments involving phage in hIgG from Figure S1.
